# tomoseqr: A Bioconductor package for spatial reconstruction and visualization of 3D gene expression patterns based on RNA tomography

**DOI:** 10.1371/journal.pone.0311296

**Published:** 2025-01-08

**Authors:** Ryosuke Matsuzawa, Daichi Kawahara, Makoto Kashima, Hiromi Hirata, Haruka Ozaki

**Affiliations:** 1 Master’s Program in Medical Sciences, Graduate School of Comprehensive Human Sciences, University of Tsukuba, Tsukuba, Ibaraki, Japan; 2 Department of Chemistry and Biological Science, College of Science and Engineering, Aoyama Gakuin University, Sagamihara, Kanagawa Japan; 3 Department of Biomolecular Science, Faculty of Science, Toho University, Funabashi, Chiba, Japan; 4 Bioinformatics Laboratory, Institute of Medicine, University of Tsukuba, Tsukuba, Ibaraki, Japan; Laboratoire Arago, FRANCE

## Abstract

RNA tomography computationally reconstructs 3D spatial gene expression patterns genome-widely from 1D tomo-seq data, generated by RNA sequencing of cryosection samples along three orthogonal axes. We developed tomoseqr, an R package designed for RNA tomography analysis of tomo-seq data, to reconstruct and visualize 3D gene expression patterns through user-friendly graphical interfaces. We show the effectiveness of tomoseqr using simulated and real tomo-seq data, validating its utility for researchers. R package tomoseqr is available on Bioconductor (https://doi.org/doi:10.18129/B9.bioc.tomoseqr) and GitHub (https://github.com/bioinfo-tsukuba/tomoseqr).

## Introduction

Understanding the spatial expression patterns of genes is pivotal for unraveling developmental processes and deciphering gene functions. RNA tomography is a computational method to reconstruct three-dimensional (3D) spatial patterns of gene expression in a genome-wide manner from one-dimensional (1D) tomo-seq data [1]. Tomo-seq data are generated by RNA sequencing of cryosections of embryos or tissue samples along three orthogonal axes (e.g., anteroposterior, dorsoventral, and left-right axes). Tomo-seq has been applied to the whole body [2], embryo [1, 3, 4], and organs [5, 6] of various animals, including humans. By utilizing tomo-seq data, RNA tomography provides 3D spatial expression patterns of myriad genes simultaneously across diverse biological and clinical samples.

Several tools were developed to analyze tomo-seq data (Table 1). Most of these tools are limited to reconstructing 1D or 2D spatial gene expression patterns and do not provide support for the 3D reconstruction of gene expression patterns using RNA tomography (TomoQC [7]; tomographer [8]; tomoda [9]). Furthermore, despite Junker et al.’s pioneering work on RNA tomography and their 3D expression pattern reconstructions, their visualization was constrained to continuous 2D cross-sections, and no publicly available tools enabled comprehensive 3D visualization for RNA tomography.

**Table 1:** Comparison with other tools.

Functions	TomoQC [7]	tomoda [9]	tomographer [8]	MATLAB script [1]	tomoseqr
Language	R	R	Python	MATLAB	R
Check of oversequencing	○	-	-	-	-
Spike-in ratio	○	-	-	-	-
Detection of genes unique to each section	○	-	-	-	-
QC	○	-	-	-	-
Normalization	-	○	-	-	-
Scaling	-	○	-	-	-
Correlation analysis (intersectional)	-	○	-	-	-
Dimension reduction methods	-	○	-	-	-
Clustering	-	○	-	-	-
Peak genes detection	-	○	-	-	-
Dimension reduction only on peak genes	-	○	-	-	-
Heatmap	-	○	-	-	-
Correlation analysis (intergene)	-	○	-	-	-
Plotting 1D expression pattern	-	○	-	-	○
2D reconstruction of expression pattern	-	-	○	-	-
Plotting 2D expression pattern	-	-	○	-	-
3D reconstruction of expression pattern	-	-	-	○	○
Plotting 3D expression pattern	-	-	-	-	○
Interactive mask generation interface	-	-	-	-	○
Interactive plot for 3D expression pattern	-	-	-	-	○

**Fig 1:**
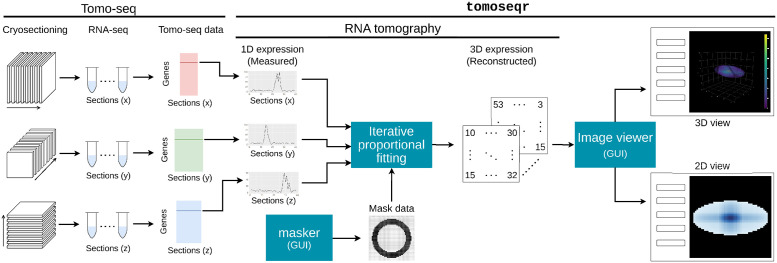
Overview of tomoseqr. Based on 1D tomo-seq data along three mutually orthogonal axes and a mask data, tomoseqr performs RNA tomography using iterative proportional fitting and reconstructs a 3D expression pattern of each gene. A GUI called *masker* helps users to design and edit a mask data. Another GUI *Image viewer* visualizes the reconstructed gene expression patterns in 2D or 3D view.

Here, we developed the R package tomoseqr, which is designed to reconstruct 3D spatial gene expression patterns from 1D tomo-seq data along three orthogonal axes using RNA tomography (Fig 1). It also includes an interactive graphical user interface (GUI) for easy visualization of the 3D patterns. We tested the capabilities of tomoseqr with simulated and real tomo-seq data from zebrafish and planarian samples, showing that tomoseqr can efficiently reconstruct and visualize the 3D spatial distribution of gene expression.

## Materials and methods

### Overview of tomoseqr

tomoseqr consists of three functionalities for RNA tomography (Fig 1). First, tomoseqr provides *masker*, the graphical user interface (GUI) to create a mask data (see below). Second, tomoseqr applies iterative proportional fitting to 1D tomo-seq data along three mutually orthogonal axes and the mask data, resulting in reconstructing the 3D expression pattern of a gene. Third, tomoseqr visualizes the expression patterns in 2D or 3D view using the graphical user interface (GUI) *Image viewer*.

### Reconstruction algorithm for RNA tomography

The iterative proportional fitting (IPF) is a statistical method to adjust the elements of a multidimensional matrix to match specific marginal totals for each axis while preserving the matrix’s overall structure [10]. tomoseqr employs IPF, which was also used in the original study of RNA tomography [1], to reconstruct the 3D pattern of gene expression from 1D tomo-seq data along three mutually orthogonal axes (usually corresponding to the anterior-posterior, ventral-dorsal, and left-right axes). Briefly, IPF iteratively (1) fits the marginal distribution of the tentative reconstructed 3D expression distribution of a gene to its 1D distribution in 1D tomo-seq data along each axis and (2) updates the reconstructed 3D gene expression distribution.

#### Problem definition

We assume the preprocessed tomo-seq data derived from *l*_*x*_, *l*_*y*_, and *l*_*z*_ cryosections along *x*-, *y*-, and *z*-axes, respectively. Let ***X***_*g*_, ***Y***_*g*_, and ***Z***_*g*_ be *l*_*x*_-, *l*_*y*_-, and *l*_*z*_-length vectors representing the expression level of a gene *g* in the preprocessed tomo-seq data along *x*-, *y*-, and *z*-axes, respectively.

Let $R_{g}^{\left(t\right)}$ be a *l*_*x*_ × *l*_*y*_ × *l*_*z*_ array that holds the reconstruction result for a gene *g*, where *t* is the number of updates. The element ${R_{g}^{\left(t\right)}}_{i,j,k}$ (1 ≤ *i* ≤ *l*_*x*_, 1 ≤ *j* ≤ *l*_*y*_, 1 ≤ *k* ≤ *l*_*z*_) represents the reconstructed gene expression level of a voxel at a position (*i*, *j*, *k*).

Our goal is to reconstruct 3D expression patterns $R_{g}^{\left(t\right)}$ based on ***X***_*g*_, ***Y***_*g*_, and ***Z***_*g*_ for each gene *g*. The loss function of reconstruction error $L(R_{g}^{\left(t\right)},X_{g},Y_{g},Z_{g})$ is described below.

#### Mask data

Mask data represents the region where the sample material is located across the *l*_*x*_ × *l*_*y*_ × *l*_*z*_ voxels in $R_{g}^{\left(t\right)}$. Let a mask data ***M*** be a *l*_*x*_ × *l*_*y*_ × *l*_*z*_ array whose element is 0 or 1. The value of each element of the mask *M*_*i*,*j*, *k*_ is 1 if the element corresponds to the coordinate at which the sample material is located; otherwise 0.

We note that the use of mask data is essential for accurately reconstructing the 3D distribution of gene expression using the IPF algorithm. Specifically, the mask defines the voxels where gene expression is reasonably expected (or not expected) in the original sample, based on prior knowledge or assumptions about the sample’s morphology. Without a mask, the IPF algorithm would distribute the 1D gene expression data (i.e., the marginal distributions along each axis) across the entire 3D space in a manner that may not be reasonable. The mask ensures that the reconstructed 3D distribution aligns with the expected regions of gene expression, thereby improving reconstruction accuracy. In other words, by predefining the allocation of expression to specific areas, the mask helps reduce artifacts that could arise during the reconstruction process.

#### IPF algorithm

1. Use mask data as the initial value $R_{g}^{\left(0\right)}$ of the reconstruction array $R_{g}^{\left(t\right)}$ at *t* = 0. This prevents the assignment of expression levels to voxels without samples, thereby reducing artifacts.
2. Calculate $R_{g}^{\left(1\right)}$ using $R_{g}^{\left(0\right)}$ and tomo-seq data ***X***_*g*_ along the *x* axis. Each element of $R_{g}^{\left(1\right)}$ is calculated as follows:
  $$\begin{array}{c} {R_{g}^{\left(1\right)}}_{i,j,k}={R_{g}^{\left(0\right)}}_{i,j,k}\cdot \frac{X_{g}\left(i\right)}{\sum_{j^{\prime }k^{\prime }}{R_{g}^{\left(0\right)}}_{i,j^{\prime },k^{\prime }}} \end{array}$$ (1)
  This operation makes $\sum_{j^{\prime }k^{\prime }}{R_{g}^{\left(1\right)}}_{i,j^{\prime },k^{\prime }}$ equal to *X*_*g*_(*i*).
3. Calculate $R_{g}^{\left(2\right)}$ using $R_{g}^{\left(1\right)}$ and tomo-seq data ***Y***_*g*_ along the *y* axis. Each element of $R_{g}^{\left(2\right)}$ is calculated as follows:
  $$\begin{array}{c} {R_{g}^{\left(2\right)}}_{i,j,k}={R_{g}^{\left(1\right)}}_{i,j,k}\cdot \frac{Y_{g}\left(j\right)}{\sum_{i^{\prime }k^{\prime }}{R_{g}^{\left(1\right)}}_{i^{\prime },j,k^{\prime }}} \end{array}$$ (2)
  This operation makes $\sum_{i^{\prime }k^{\prime }}{R_{g}^{\left(2\right)}}_{i^{\prime },j,k^{\prime }}$ equal to *Y*_*g*_(*j*).
4. Calculate $R_{g}^{\left(3\right)}$ using $R_{g}^{\left(2\right)}$ and tomo-seq data ***Z***_*g*_ along the *z* axis. Each element of $R_{g}^{\left(3\right)}$ is calculated as follows:
  $$\begin{array}{c} {R_{g}^{\left(3\right)}}_{i,j,k}={R_{g}^{\left(2\right)}}_{i,j,k}\cdot \frac{Z_{g}\left(k\right)}{\sum_{i^{\prime }j^{\prime }}{R_{g}^{\left(2\right)}}_{i^{\prime },j^{\prime },k}} \end{array}$$ (3)
  This operation makes $\sum_{i^{\prime }j^{\prime }}{R_{g}^{\left(3\right)}}_{i^{\prime },j^{\prime },k}$ equal to *Z*_*g*_(*k*).
5. Repeat steps 2 through 4 for a predetermined number of times *m* (*m* = 100 by default).
6. The loss function $L(R_{g}^{\left(t\right)},X_{g},Y_{g},Z_{g})$ after *m* sets of iterations (with *t* = 3*m* updates completed) is as follows:
  $$\begin{array}{c} L(R_{g}^{\left(t\right)},X_{g},Y_{g},Z_{g})=\sum_{i}{\left(\sum_{j^{\prime }k^{\prime }}{R_{g}^{\left(3m\right)}}_{i,j^{\prime },k^{\prime }}-X_{g}\left(i\right)\right)}^{2}+ \end{array}$$ (4)
  $$\begin{array}{cc} & \sum_{j}{\left(\sum_{i^{\prime }k^{\prime }}{R_{g}^{\left(3m\right)}}_{i^{\prime },j,k^{\prime }}-Y_{g}\left(j\right)\right)}^{2}+ \end{array}$$ (5)
  $$\begin{array}{cc} & \sum_{k}{\left(\sum_{i^{\prime }j^{\prime }}{R_{g}^{\left(3m\right)}}_{i^{\prime },j^{\prime },k}-Z_{g}\left(k\right)\right)}^{2} \end{array}$$ (6)

### Preprocessing of tomo-seq data

Before applying IPF, tomoseqr preprocess 1D tomo-seq data.

Let *n* be the number of genes and *l*_*x*_, *l*_*y*_, and *l*_*z*_ be the number of cryosections along *x*-, *y*-, and *z*- axes, respectively. Let ***X*** be a *n* × *l*_*x*_ matrix that represents 1D tomo-seq data for *x*-axis. Let ***Y*** be a *n* × *l*_*y*_ matrix that represents 1D tomo-seq data for *y*-axis. Let ***Z*** be a *n* × *l*_*z*_ matrix that represents 1D tomo-seq data for *z*-axis.

Let ***M*** be the mask array, which is an *l*_*x*_ × *l*_*y*_ × *l*_*z*_ array whose element is 0 or 1. The value of each element of the mask *M*_*i*,*j*, *k*_ is 1 if the element corresponds to the coordinate at which the sample material is located; otherwise 0.

The tomo-seq data is preprocessed as follows:

1. The sum of elements of ***M*** is calculated for each section as follows:
  $$\begin{array}{c} m^{\left(x\right)}=(\begin{array}{c} \sum_{j=1}^{l_{y}}\;\sum_{k=1}^{l_{z}}M_{1,j,k},\ldots ,\sum_{j=1}^{l_{y}}\;\sum_{k=1}^{l_{z}}M_{l_{x},j,k} \end{array}) \end{array}$$ (7)
  $$\begin{array}{c} m^{\left(y\right)}=(\begin{array}{c} \sum_{i=1}^{l_{x}}\;\sum_{k=1}^{l_{z}}M_{i,1,k},\ldots ,\sum_{i=1}^{l_{x}}\;\sum_{k=1}^{l_{z}}M_{i,l_{y},k} \end{array}) \end{array}$$ (8)
  $$\begin{array}{c} m^{\left(z\right)}=(\begin{array}{c} \sum_{i=1}^{l_{x}}\;\sum_{j=1}^{l_{y}}M_{i,j,1},\ldots ,\sum_{i=1}^{l_{x}}\;\sum_{j=1}^{l_{y}}M_{i,j,l_{z}} \end{array}) \end{array}$$ (9)
  where ***m***^(***x***)^, ***m***^(***y***)^, and ***m***^(***z***)^ are *l*_*x*_-, *l*_*y*_-, and *l*_*z*_-length vectors, respectively, and represent the relative sample material volume per section.
2. Let *g* be the index of a gene of interest. To normalize the total gene expression per sample material volume across sections, the inter-section normalization option is applied to ${\left\{X_{g,i}\right\}}_{i=1}^{l_{x}}$, ${\left\{Y_{g,j}\right\}}_{j=1}^{l_{y}}$, and ${\left\{X_{g,k}\right\}}_{k=1}^{l_{z}}$ (i.e., the *g*-th row vectors of ***X***, ***Y***, and ***Z***, respectively). Specifically, for each section, each element is divided by the total expression and multiplied by the relative sample material volumes as follows:
  $$\begin{array}{c} X_{g}^{\prime }=(\begin{array}{c} \frac{X_{g,1}\cdot m_{1}^{\left(x\right)}}{\sum_{h=1}^{n}X_{h,1}},\cdots ,\frac{X_{g,l_{x}}\cdot m_{l_{x}}^{\left(x\right)}}{\sum_{h=1}^{n}X_{h,l_{x}}} \end{array}) \end{array}$$ (10)
  $$\begin{array}{c} Y_{g}^{\prime }=(\begin{array}{c} \frac{Y_{g,1}\cdot m_{1}^{\left(y\right)}}{\sum_{h=1}^{n}Y_{h,1}},\cdots ,\frac{Y_{g,l_{y}}\cdot m_{l_{y}}^{\left(y\right)}}{\sum_{h=1}^{n}Y_{h,l_{y}}} \end{array}) \end{array}$$ (11)
  $$\begin{array}{c} Z_{g}^{\prime }=(\begin{array}{c} \frac{Z_{g,1}\cdot m_{1}^{\left(z\right)}}{\sum_{h=1}^{n}Z_{h,1}},\cdots ,\frac{Z_{g,l_{z}}\cdot m_{l_{z}}^{\left(z\right)}}{\sum_{h=1}^{n}Z_{h,l_{z}}} \end{array}) \end{array}$$ (12)
  where $X_{g}^{\prime },Y_{g}^{\prime },Z_{g}^{\prime }$ are the normalized *g*-th row vectors.
3. The sum of expression levels for each of ***X***, ***Y***, and ***Z*** are averaged as *T*:
  $$\begin{array}{c} T=\frac{\sum_{j=1}^{l_{x}}\sum_{i=1}^{n}X_{i,j}+\sum_{j=1}^{l_{y}}\sum_{i=1}^{n}Y_{i,j}+\sum_{j=1}^{l_{z}}\sum_{i=1}^{n}Z_{i,j}}{3} \end{array}$$ (13)
4. Each of $X_{g}^{\prime }$, $Y_{g}^{\prime }$, and $Z_{g}^{\prime }$ is divided by its own row sum and then multiplied by *T*. Let $X_{g}^{\prime \prime }$, $Y_{g}^{\prime \prime }$, and $Z_{g}^{\prime \prime }$ as the resultant preprocessed vectors as follows:
  $$\begin{array}{cc} X_{g}^{\prime \prime } & =\frac{T}{\sum_{i=1}^{l_{x}}X_{g,i}^{\prime }}X_{g}^{\prime } \end{array}$$ (14)
  $$\begin{array}{cc} Y_{g}^{\prime \prime } & =\frac{T}{\sum_{j=1}^{l_{y}}Y_{g,j}^{\prime }}Y_{g}^{\prime } \end{array}$$ (15)
  $$\begin{array}{cc} Z_{g}^{\prime \prime } & =\frac{T}{\sum_{k=1}^{l_{z}}Z_{g,k}^{\prime }}Z_{g}^{\prime } \end{array}$$ (16)

IPF algorithm is performed on $X_{g}^{\prime \prime }$, $Y_{g}^{\prime \prime }$, and $Z_{g}^{\prime \prime }$.

### Masker

**Fig 2:**
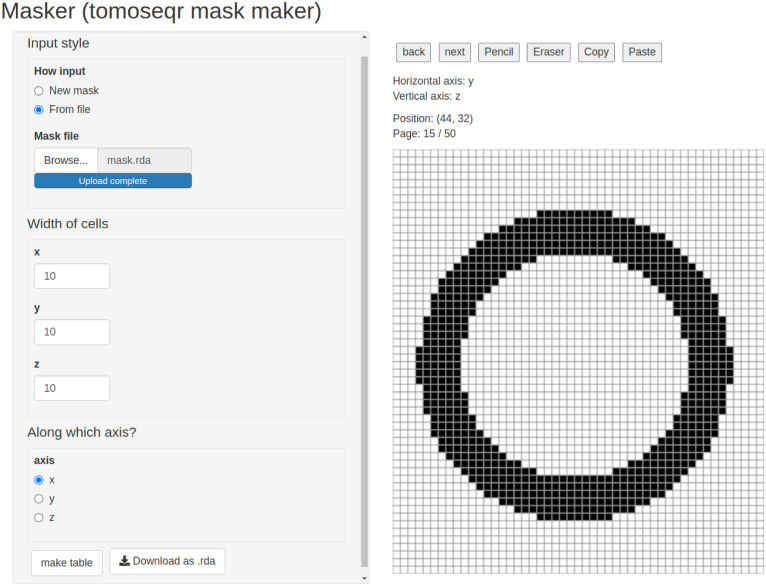
User interface of *masker*.

tomoseqr requires “mask” data in addition to tomo-seq data to reconstruct expression patterns. Mask data is a three-dimensional binary array that defines the shape of the sample material from which tomo-seq data was derived (as described in the subsection “Mask data” above). *Masker* is the Shiny-based GUI that enables users to create mask data by drawing a mask as a 2D image of a sample material for each section along the user-defined axis (Fig 2 and S1 Video). Mask data created using *masker* can be exported as an R Data File.

### Image viewer

**Fig 3:**
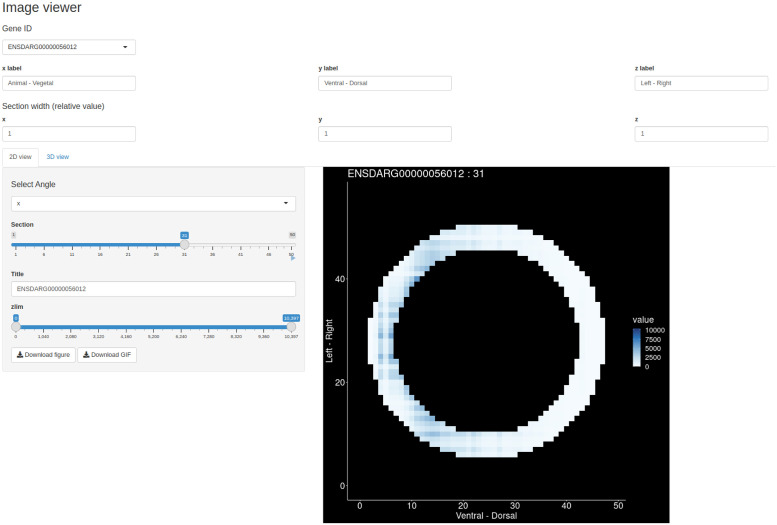
User interface of *Image viewer* (2D view).

**Fig 4:**
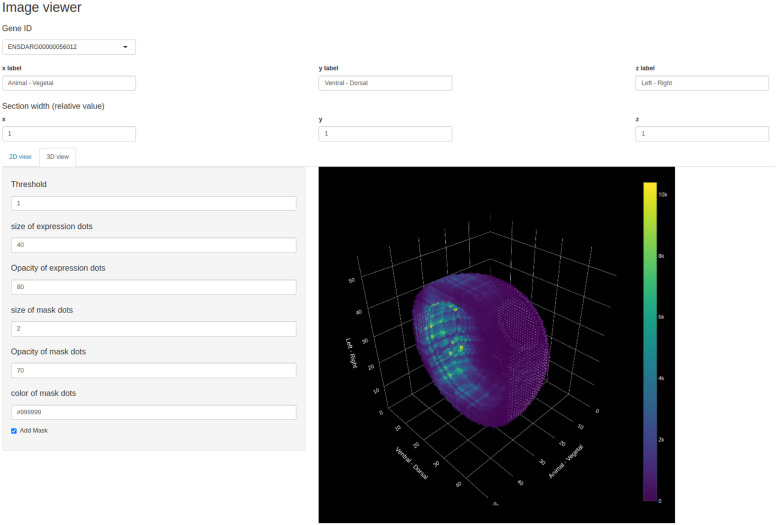
User interface of *Image viewer* (3D view).

Since tomoseqr’s output is a 3D array whose elements are the reconstructed expression levels for a gene, visualization is essential to interpret the reconstruction result. *Image viewer* is the Shiny-based GUI that allows users to interactively visualize 2D cross-section tomographic images and 3D plots of the reconstruction results. In the 2D view, the gene expression pattern is visualized as a heat map in each cross section orthogonal to each axis (Fig 3 and S2 Video). In the 3D view, the gene expression pattern is visualized as an interactive 3D plot (Fig 4 and S3 Video). *Image viewer* can output 2D tomographic images in PNG and GIF format and 3D images in PNG format.

### Implementation and code availability of tomoseqr

tomoseqr was implemented as an R package and is available on Bioconductor (https://doi.org/doi:10.18129/B9.bioc.tomoseqr) and GitHub (https://github.com/bioinfo-tsukuba/tomoseqr).

### Evaluation using simulated tomo-seq data

#### Generation of simulated tomo-seq data

**Fig 5:**
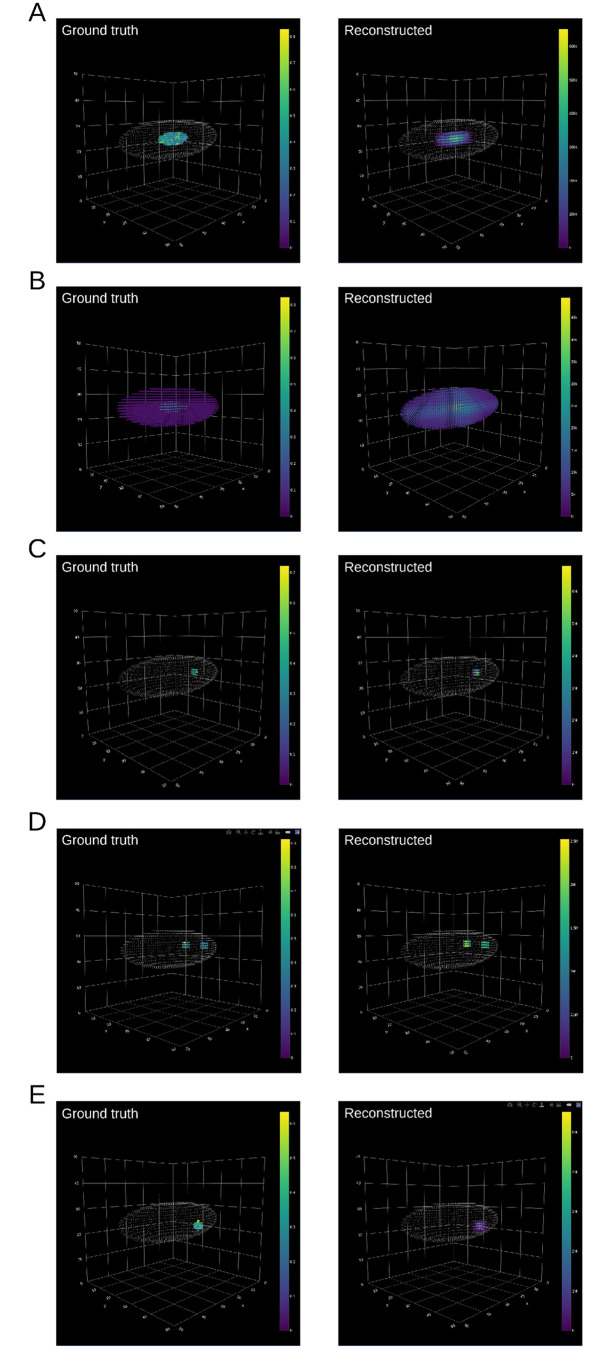
Spatial expression patterns and reconstruction results for Gene1, Gene2, Gene3, Gene4, and Gene5. Ground truth and reconstruction results are shown for (A) Gene1, (B) Gene2, (C) Gene3, (D) Gene4, and (E) Gene5. The dots are colored by gene expression levels, with white dots representing the sample shape (mask).

To evaluate the reconstruction algorithm in tomoseqr, we generated simulated tomo-seq data. In the simulation, we assumed that the sample material was a ellipsoid and 50 cryosections were prepared for each of the three orthogonal axes. Accordingly, tomoseqr was applied to the simulated tomo-seq data to reconstruct gene expression patterns across the voxels within the ellipsoid (i.e., mask is 1) out of 50 × 50 × 50 voxels. We also assumed that the simulated tomo-seq data consisted of genes that exhibit spatially specific patterns (spatial genes) and those that do not (background genes). We set the number of spatial genes to 3 and that of background genes to 1,997 or 4,997 and generated the simulated tomo-seq data as follows (Fig 5):

1. For each of the spatial and background genes, we first generated a simulated 3D expression array {*E*_*i*,*j*, *k*_} (*i*, *j*, *k* are the indexes of cryosections for the three orthogonal axes). For spatial genes, we generated 3D expression arrays for three spatial genes (Gene1, Gene2, Gene3) with the following 3D gene expression patterns (Fig 5):
  **Gene1** Strongly expressed in the center of the ellipsoid and no expression in other regions.
  **Gene2** Strongly expressed in the center of the ellipsoid, weakly expressed in other regions.
  **Gene3** Strongly expressed in a narrow region in front of the ellipsoid, with no expression in other regions.
  **Gene4** Strongly expressed in a symmetrical narrow region in front of the ellipsoid, with no expression in other regions.
  **Gene5** Strongly expressed in a narrow region located away from the left-right axis and the dorsal-ventral axis, with no expression in other regions.
  For background genes, 3D gene expression arrays were generated for 1,997 or 4,997 genes using the SPsimSeq package [11].
2. For each gene, the 3D expression pattern array was converted to 1D tomo-seq data for each axis. Specifically, we calculated the marginal sum of {*E*_*i*,*j*, *k*_} over two of the three axes while preserving the remaining one:
  $$\begin{array}{cc} {\text{x}}_{i} & =\sum_{j}\;\sum_{k}E_{i,j,k} \end{array}$$
  $$\begin{array}{cc} {\text{y}}_{j} & =\sum_{i}\;\sum_{k}E_{i,j,k} \end{array}$$
  $$\begin{array}{cc} {\text{z}}_{k} & =\sum_{j}\;\sum_{i}E_{i,j,k} \end{array}$$
  This result in the simulated 1D tomo-seq data for each gene along the three axes: (x_1_, x_2_, …, x_50_), (y_1_, y_2_, …, y_50_), (z_1_, z_2_, …, z_50_).
3. For each of the three axes, simulated 1D tomo-seq data were combined across the genes. This results in simulated tomo-seq data of 2,000 or 5,000 genes and 50 cryosections along the axis.
4. We repeated the above procedures 10 times, resulting in 10 simulated tomo-seq data with 2,000 and 5,000 genes.

#### Accuracy evaluation of tomoseqr using simulation data

tomoseqr was run on the simulated tomo-seq data with the inter-section normalization option. The accuracy of gene expression pattern reconstructions by tomoseqr was evaluated using the simulated tomo-seq data. Specifically, we calculated the Pearson’s correlation coefficient (PCC) between the reconstructed result and the ground truth. For each gene, the ground truth was defined as the gene expression level normalized to reads per million (RPM) in each voxel.

To verify the significance of the PCCs between the reconstruction results and ground truths, a randomization test was conducted. In the test, a randomized array was generated by randomly shuffling the position of each element of the reconstruction result. Then, the PCC between the randomized array and the ground truth was calculated. Note that the elements with a mask value of 0, i.e., voxels not included in the sample, were excluded from both the shuffle of elements and the calculation of PCCs. The above operations were performed 1,000 times for each of the 10 simulated tomo-seq data for each spatial gene to obtain the empirical null distribution of the PCCs. Finally, PCCs of the 10 simulated tomo-seq data and the empirical null distribution aggregated across the 10 simulated tomo-seq data were compared using the Kolmogorov-Smirnov test.

### Evaluation of computation time and memory usage

We conducted a computer experiment using the zebrafish (*Danio rerio*) tomo-seq data [1] to evaluate the amount of computing resources used by tomoseqr during its execution. We prepared the tomo-seq data from the zebrafish shield stage by converting the tables entitled traces_shield_AV.txt (for animal-vegetal (AV) axis), traces_shield_VD.txt (for ventral-dorsal (VD) axis), and traces_shield_LR.txt (for left-right (LR) axis) in the Table S4 spreadsheet in Junker *et al.* [1] into comma-separated value (CSV) files. Using tomoseqr, we reconstructed 3D expression patterns for 100, 1,000, and 2,352 genes (genes expressed with at least one section with read count > 50 in tomo-seq data for the AV axis) and measured computation time and memory usage. Computation time was measured as the time of the actual reconstruction process. The amount of memory usage was measured when the entire R script was executed. We used a computer with the following specs:

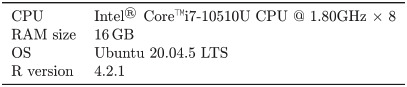


### Application of tomoseqr to zebrafish tomo-seq data

To evaluate the reconstruction performance of tomoser with real tomo-seq data, we applied tomoser on publicly available zebrafish tomo-seq data [1] and compared the reconstruction results by tomoser with previously published *in situ* hybridization results. We used the tomo-seq data from the zebrafish shield stage from Juker *et al.* [1] (as described above). A hollow hemisphere was used as mask data and the size of the hemisphere was matched to the expression distribution of the mitochondria-related gene (*mt-co2*). tomoseqr was run with inter-section normalization to normalize the total gene expression per sample volume across sections.

### Application of tomoseqr on planarian tomo-seq data

#### Maintenance of planarian

A lab stock of asexual planarians *D. japonica* purchased from Aqua Field (Tokyo, Japan) was maintained in deionized water containing 0.05 g/L Instant Ocean Sea Salt (Instant Ocean, Blacksburg, VA, USA). The planarians were fed with chicken liver once or twice a week. The intact (completely-regenerated) planarians were starved for at least one week prior to the following experiments.

#### Preparation of planarian sections followed by total RNA purification

Three planarians were anesthetized and straightened with 0.2% Chloretone Hemihydrate (Tokyo Chemical Industry Co., Ltd., Tokyo, Japan) in deionized water containing 0.05 g/L Instant Ocean Sea Salt for a minute. The straightened planarians were moved into Tissue-Tek Cryomold [10 × 10 × 5 mm] (Sakura Finetek Japan Co., Ltd., Osaka, Japan). After removing the liquid, O.C.T compound (Sakura Finetek Japan Co., Ltd., Osaka, Japan) was added, followed by freezing them using liquid nitrogen. The series of sections (78 sections along anterior-posterior axis, 28 sections along dorsal-ventral axis, and 51 sections along left-right axis) was prepared with CM3050 S (Leica, Wetzlar, German). The thickness of sections along anterior-posterior axis, dorsal-ventral axis, and left-right axis were 80 *μ*m, 20 *μ*m, and 20 *μ*m, respectively. Each section was moved into each well of the twin.tec PCR plates LoBind, semi-skirted (Eppendorf, Hamburg, German) using a chilled handled needle in the chilled CM3050 S. Total RNA was extracted from each individual of the planarians using “Direct-TRI” method [12]. Briefly, 100 *μ*L of TRI Reagent LS (Molecular Research Center, Cincinnati, OH, USA) was added to each well. After lysed the sections by vortexing, 100 *μ*L of 99.5% EtOH was added, followed by mixing well. Then, the lysate was purified using AcroPrep Advance 96-well Long Tip Filter Plate for Nucleic Acid Binding (Pall, Port Washington, NY, USA). RNA was eluted with 10 *μ*L nuclease-free water.

#### RNA-Seq library preparation and sequencing

3’ mRNA-Seq were conducted according to the Lasy-Seq ver. 1.1 protocol (https://sites.google.com/view/lasy-seq/) [13, 14]. Briefly, 9 *μ*L of the purified total RNA were reverse transcribed using an RT primer with index and SuperScript IV reverse transcriptase (Thermo Fisher Scientific, Waltham, MA, USA). Then, all RT mixtures of the samples were pooled and purified using an equal volume of AMpure XP beads (Beckman Coulter, Brea, CA, USA) according to the manufacturer’s instructions. Second strand synthesis was conducted on the pooled samples using RNaseH (5U/ L, Enzymatics, Beverly, MA, USA), and DNA polymerase I (10U/ *μ*L, Enzymatics, Beverly, MA, USA). To avoid the carryover of large amounts of rRNAs, the mixture was subjected to RNase treatment using RNase T1 (Thermo Fisher Scientific, Waltham, MA, USA). Then, purification was conducted with a 0.8 × volume of AMpure XP beads. Fragmentation, end-repair, and A-tailing were conducted using 5 × WGS Fragmentation Mix (Enzymatics, Beverly, MA, USA). The adapter for Lasy-Seq was ligated using 5 × Ligation Mix (Enzymatics, Beverly, MA, USA), and the adapter-ligated DNA was purified twice with a 0.8 × volume of AMpure XP beads. After optimisation of PCR cycles for library amplification by qPCR using Evagreen, 20 × in water (Biotium, Fremont, CA, USA) and the QuantStudio5 Real-Time PCR System (Applied Biosystems, Waltham, MA, USA), the library was amplified using KAPA HiFi HotStart ReadyMix (KAPA BIOSYSTEMS, Wilmington, MA, USA) on the ProFlex PCR System (Applied Biosystems, Waltham, MA, USA). The amplified library was purified with an equal volume of AMpure XP beads. One microliter of the library was then used for electrophoresis using a Bioanalyzer 2100 with the Agilent High Sensitivity DNA kit (Agilent Technologies, Santa Clara, CA, USA) to check for quality. Then, sequencing of 150-bp paired-end reads was performed using HiSeq X Ten (Illumina, San Diego, CA, USA).

#### Read mapping and gene expression quantification

Read 1 reads were processed with fastp (version 0.21.0) [15] using the following parameters:

--trim_poly_x

-w 20

--adapter_sequence=AGATCGGAAGAGCACACGTCTGAACTCCAGTCA

--adapter_sequence_r2=AGATCGGAAGAGCGTCGTGTAGGGAAAGAGTGT

-l 31

The trimmed reads were then mapped to a *D. japonica* reference transcriptome sequences deposited in http://www.planarian.jp/download.html [16], using BWA mem (version 0.7.17-r1188) [17] with the default parameters. The read count for each gene was calculated with Salmon using -l IU, which specifies the library type (version v0.12.0) [18].

#### Reconstruction of 3D gene expression pattern from planarian tomo-seq data

We applied tomoseqr on the planarian tomo-seq data. The mask data was created with *masker* according to the morphology of an adult planarian. tomoseqr was run with inter-section normalization to normalize the total gene expression per sample volume across sections.

### Systematic exploration of correlated and regionalized expression patterns among planarian genes

As part of our analysis of large-scale gene expression distributions, we investigated genes with expression patterns similar to those of specific target genes. Additionally, we explored genes with spatially specific expression patterns using autocorrelation methods.

#### Identification of genes with expression patterns similar to specific targets

1. We reconstructed the spatial expression distributions of genes from the tomo-seq data.
2. For each spatial expression distribution, we calculated the correlation coefficient with the spatial expression distribution of *opsin* using the correlationWithSpecificGene function. This function is available in the GitHub version of tomoseqr and will be included in Bioconductor version 3.20.
3. We obtained the reconstructed spatial expression distributions for the four genes that showed the highest correlation coefficients with *opsin*.

#### Identification of genes with spatially specific expression patterns using autocorrelation

Following Mayeur *et al*. 2021 [3], we performed an autocorrelation analysis using Moran’s index (Moran’s I). The analysis was conducted as follows:

1. We listed planarian genes that have homologs in mice. Out of the 255,666 genes analyzed, 14,576 were identified as such homologous genes.
2. We filtered the homologous genes based on the list of mouse proteins associated with GO:0001228, which means “DNA-binding transcription activator activity, RNA polymerase II-specific” [19]. As a result, 214 genes were identified.
3. We reconstructed the spatial expression distributions of planarian genes that are homologs of mouse transcription factors using tomo-seq data.
4. For each spatial expression distribution, we calculated Moran’s I.
5. We obtained the reconstructed spatial expression distributions and the names of the homologous mouse transcription factors for the five genes that exhibited the highest Moran’s I values.

## Results

### Accuracy evaluation of tomoseqr using simulated data

The accuracy of gene expression pattern reconstructions by tomoseqr was evaluated using the simulation data (Materials and methods). We employed two simulation settings, in which the expressions of 2,000 (3 spatial and 1,997 background genes) or 5,000 (3 spatial and 4,997 background genes) genes were considered, and generated 10 simulated datasets for each simulation setting. Subsequently, tomoseqr was applied to each of the simulated datasets with the inter-section normalization option, and the reconstruction results of 3 spatial genes (Gene1, Gene2, and Gene3) were compared with the ground truth patterns by the Pearson’s correlation coefficients (PCCs).

Fig 5 shows representative results of the reconstructed gene expression patterns of 5 spatial genes (Gene1, Gene2, Gene3, Gene4 and Gene5). The reconstruction results for each gene were consistent with the ground truth. Table 2 shows the PCCs of reconstruction results and ground truths for all simulated datasets. All of the spatial genes showed high PCC values in both simulation settings while the reconstruction results for Gene2 showed relatively moderate PCCs compared to other genes.

**Table 2:** Evaluation of the reconstruction accuracy of tomoseq using simulated tomo-seq data. For each simulating setting and spatial gene, 10 simulated tomo-seq data were generated. tomoseqr was applied to these data with the inter-section normalization option. Means and standard deviations of PCCs of the reconstruction results with the ground truth across 10 simulated tomo-seq data are shown. P-values were calculated by randomly shuffling the reconstructed results and calculating PCCs of the randomized results with the ground truth 1,000 times, following Kolmogorov-Smirnov tests (Materials and methods).

Simulation setting	Spatial gene	PCC (with ground truth)	p-value
2000 genes	Gene1	0.849 ± 0.00259	< 5.03 × 10^−9^
	Gene2	0.641 ± 0.00314	< 5.03 × 10^−9^
	Gene3	0.910 ± 0.0236	< 5.03 × 10^−9^
	Gene4	0.969 ± 0.00858	< 5.03 × 10^−9^
	Gene5	0.861 ± 0.0154	< 5.03 × 10^−9^
5000 genes	Gene1	0.847 ± 0.00402	< 5.03 × 10^−9^
	Gene2	0.638 ± 0.00375	< 5.03 × 10^−9^
	Gene3	0.914 ± 0.0173	< 5.03 × 10^−9^
	Gene4	0.969 ± 0.00530	< 5.03 × 10^−9^
	Gene5	0.861 ± 0.0153	< 5.03 × 10^−9^

To verify the significance of the PCCs between the reconstruction results and ground truths, a randomization test was conducted (Materials and methods). The observed PCCs showed statistically significantly larger values than the respective null distributions for all cases (*p* < 5.03 × 10^−9^, Kolmogorov-Smirnov test) (Table 2). These results indicate that the spatial pattern of gene expression by tomoseqr can be reconstructed with good accuracy.

### Evaluation of computation time and memory usage

We performed a computer experiment to evaluate the amount of computing resources used by tomoseqr during its execution of the reconstruction of 3D gene expression. Specifically, we run tomoseqr for 100, 1,000, and 2,352 genes on a typical laptop PC (Materials and methods).

The measurement results are shown in Table 3. The computation time was 132.7, 1,330, and 2,626 s for 100, 1,000, and 2,352 genes, respectively, suggesting that the computation time is proportional to the number of genes to be reconstructed. The amount of memory usage was 380 MB, 1.96 GB, and 3.94 GB for 100, 1,000, and 2,352 genes. This result implies that the increase in maximum memory usage is more gradual than proportional to the number of genes. These results indicate that tomoseqr can reconstruct thousands of genes with realistic computation times and maximum memory usage on a typical laptop PC.

**Table 3:** Computation time and amount of memory usage for reconstruction using tomoseqr.

Number of genes	Computation time	Memory usage
100	132.7 s	380 MB
1000	1330 s	1.96 GB
2352	2626 s	3.94 GB

### Application of tomoseqr to zebrafish tomo-seq data

To evaluate the reconstruction performance of tomoser with real tomo-seq data, we applied tomoser on publicly available zebrafish (*Danio rerio*) tomo-seq data [1] and compared the reconstruction results of tomoser with previously published *in situ* hybridization results.

**Fig 6:**
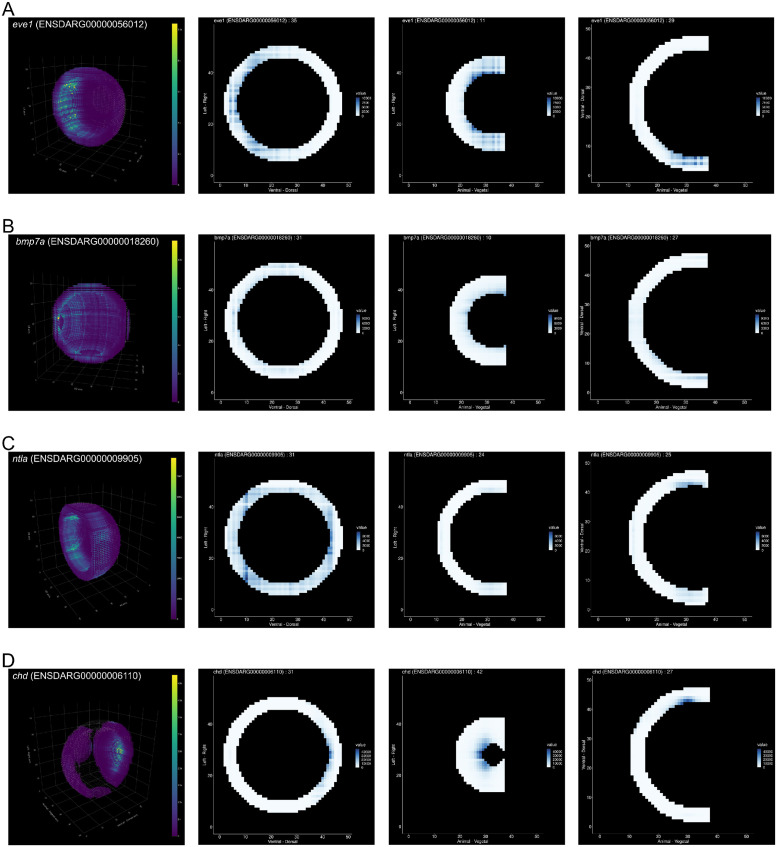
Results of reconstruction with zebrafish (*Danio rerio*, shield stage). Reconstruction result for expression of (A) *eve1* (*ENSDARG00000056012*), (B) *bmp7a* (*ENSDARG00000018260*), (C) *ntla* (*ENSDARG00000009905*), and (D) *chd* (*ENSDARG00000006110*). For each of the genes, 3D view, cross section perpendicular to the animal-vegetal axis (18 m × 50 sections), cross section perpendicular to the dorsal-ventral axis (18 m × 49 sections), and cross section perpendicular to the left-right axis (18 m × 56 sections) are shown from left to right. Parentheses after gene names indicate gene IDs. Colors represent the reconstructed expression levels.

The results are shown in Fig 6.*eve1* was strongly expressed in the ventral and near the center of the animal-vegetal (AV) axis (Fig 6A).*bmp7a* showed expression throughout the ventral side (Fig 6B).*ntla* showed expression near the center of the AV axis (Fig 6C).*chd* showed strong expression in a narrow dorsal region (Fig 6D). All of these results were consistent with previous studies using *in situ* hybridization [20–23], showing that tomoseqr can reconstruct 3D expression patterns of developmentally important genes.

### Application of tomoseqr on planarian tomo-seq data

We further applied tomoseqr on a newly generated tomo-seq data of planarian (*Dugesia japonica*). The tomo-seq data was generated by performing Lasy-Seq on the 78 sections along anterior-posterior axis, 28 sections along dorsal-ventral axis, and 51 sections along left-right axis (Materials and methods).

**Fig 7:**
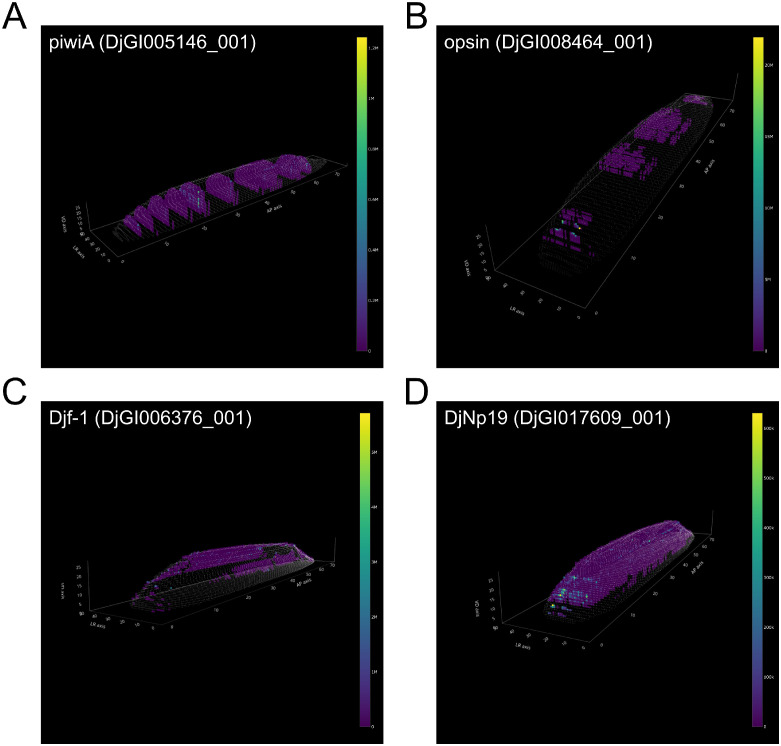
Reconstruction results using planarian tomo-seq data. Reconstruction result for expression of (A) *piwiA* (*DjGI005146_001*), (B) *opsin* (*DjGI008464_001*), (C) *Djf-1* (*DjGI006376_001*), and (D) *DjNp19* (*DjGI017609_001*). Parentheses after gene names indicate gene IDs. Colors represent the reconstructed expression levels.AP means anterior-posterior axis (80 *μ*m × 78 sections), VD means ventral-dorsal axis,(20 *μ*m × 28 sections) and LR means left-right axis (20 *μ*m × 51 sections).

Fig 7 shows the results of reconstruction with inter-section normalization. For *piwiA*, which is known to be expressed in the whole body [24], expression was confirmed throughout the body (Fig 7A).*opsin*, which is known to be expressed in the eye [25], was found in two high expression spots on the head (Fig 7B).*Djf-1* was found to be expressed in the epidermis (Fig 7C and S4 Video), which was consistent with a previous study [26].*DjNp19*, which is known to be expressed in the brain and nervous [27], expression was observed throughout the body, but was particularly strong in the head (Fig 7D and S5 Video). These results support the performance of tomoseqr to reconstruct 3D spatial gene expression patterns.

### Systematic exploration of correlated and regionalized expression patterns among planarian genes

Finally, we reconstructed the 3D spatial expression patterns of 18,768 expressed planarian genes using tomoseqr. This necessitates prioritizing genes that exhibit biologically interesting expression patterns from the large number of genes. To achieve this, we adopted two strategies: correlation and autocorrelation.

**Fig 8:**
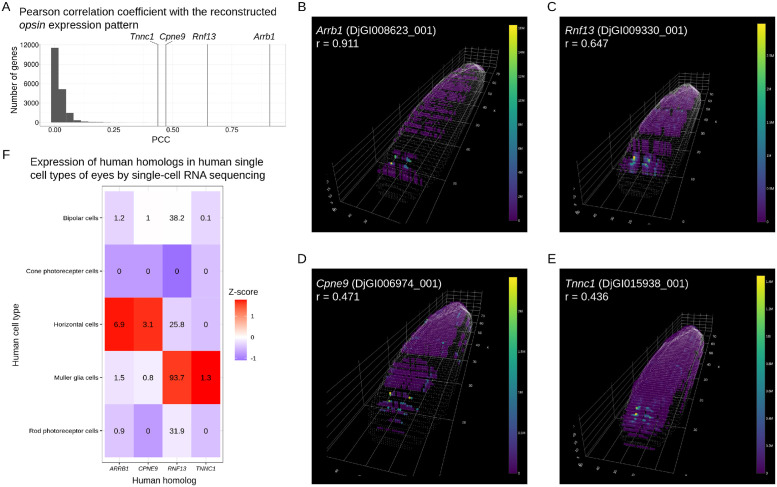
Systematic exploration of genes correlated with the reconstructed *opsin* expression pattern in planarian tomo-seq data. (A) A histogram showing the Pearson correlation coefficients (PCCs) with the reconstructed *opsin* expression pattern for 18,768 expressed genes. The vertical lines indicate the top 4 genes with the highest PCCs. (B-E) Reconstruction results for the expression of (B) *Arrb1* (*DjGI008623_001*), (C) *Rnf13* (*DjGI009330_001*), (D) *Cpne9* (*DjGI006974_001*), and (E) *Tnnc1* (*DjGI015938_001*). The PCCs of these gene expression patterns with the *opsin* gene (Fig 7B) are shown. (F) A heatmap showing the expression of human homologs in human eye single-cell types using single-cell RNA sequencing data from the Human Protein Atlas. The gene expression levels, in normalized transcript per million (nTPM), were transformed into Z-scores.

First, we explored genes that showed a high correlation with specific spatial patterns. For instance, we identified genes specifically expressed in the eye by calculating the Pearson correlation coefficients between the reconstructed gene expression patterns and the eye-specific *opsin* gene (Fig 8A). The top four genes with the highest correlation coefficients with *opsin* all exhibited eye-specific expression (Fig 8B–8E). *Arrb1* is involved in melanopsin signaling in the mammalian retina [28], and *Rnf13* is an E3 ubiquitin-protein ligase [29] and one of the retinal pigment epithelium signature genes in rodents and humans [30]. On the other hand, although *Cpne9*, a calcium-dependent phospholipid-binding protein [31], and *Tnnc1*, a calcium-binding protein involved in muscle contraction [32], are not directly linked to visual function, they may play a role in calcium-dependent signaling relevant to vision.

**Fig 9:**
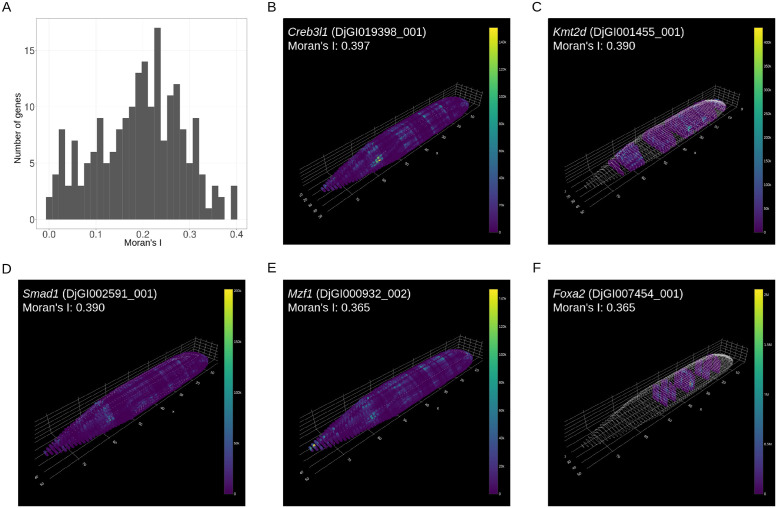
Systematic exploration of genes with spatial autocorrelation in the reconstructed expression patterns in planarian tomo-seq data. (A) A histogram showing the Moran’s I values of the reconstructed expression patterns for 214 genes encoding planarian homologs of mouse transcription factors. (B-F) Reconstruction results for the expression of (B) *Creb3l1* (*DjGI019398_001*), (C) *Kmt2d* (*DjGI001455_001*), (D) *Smad1* (*DjGI002591_001*), (E) *Mzf1* (*DjGI000932_002*), and (F) *Foxa2* (*DjGI007454_001*). The corresponding Moran’s I values are shown.

Second, we searched for genes displaying regionalized expression patterns using Moran’s index (Moran’s I), a spatial autocorrelation measure [3]. Specifically, we focused on 214 expressed genes that encode the planarian homologs of mouse transcription factors and calculated Moran’s I for these genes (Fig 9A). The top five genes with the highest Moran’s I each exhibited distinct expression patterns (Fig 9B–9F), suggesting potentially diverse functions in planarians. Consistently, the homologs of these five regionalized genes are associated with various developmental, homeostatic, and disease processes in mammals (e.g., *Creb3l1* with osteogenesis imperfecta [33], *Kmt2d* with Kabuki syndrome [34], *Smad1* with the control of cell fate [35], *Mzf1* with keratinocyte differentiation [36], and *Foxa2* with cholestatic syndromes [37]).

Together, these results demonstrate that tomoseqr, by reconstructing 3D spatial gene expression patterns, not only facilitates the investigation of known gene expression patterns but also aids in the discovery of genes with biologically significant spatial distributions, through both correlation and autocorrelation analyses.

## Discussion

Our study presents tomoseqr, a Bioconductor R package accurately and efficiently reconstructs 3D gene expression patterns using 1D tomo-seq data along three orthogonal axes. Through validation with both simulated and real datasets, including zebrafish and planarian data, we demonstrated that tomoseqr can reliably reconstruct spatial gene expression for thousands of genes.

The inclusion of Shiny-based graphical user interfaces, *masker*, and *Image viewer*, further underscores tomoseqr’s utility by providing intuitive tools for mask data creation and visualization of gene expression patterns. This makes tomoseqr accessible to a broad range of researchers, including those without extensive computational expertise.

Our work further underlines the critical role of software packages in interpreting and visualizing complex gene expression data in life sciences. As high-throughput multi-sample RNA-seq technologies become increasingly accessible and lower the barrier to RNA tomography [38], the importance of our work is set to escalate.

Once a large number of reconstructed 3D spatial expression patterns of genes are obtained using tomoseqr, it becomes crucial to explore and prioritize those with biological significance for discovery and hypothesis generation. In this study, we demonstrated that by leveraging correlation and spatial autocorrelation metrics, we can systematically identify genes with intriguing expression patterns in planarians, a species for which prior knowledge is limited. This data-driven approach to gene exploration highlights the potential of tomoseqr to significantly contribute to a wide range of research areas in the life sciences.

There are several possible directions for improving tomoseqr. The first is the improvements to the reconstruction algorithm. Although the IPF algorithm used in tomoseqr is based on numerical reconstruction, it could be possible to devise other reconstruction algorithms by incorporating biological properties, such as spatial autocorrelation, gene-gene correlation, and noise structures. The second is the enhancement of robustness through the preprocessing of tomo-seq data. In RNA-seq data, gene expression values might happen to drop to zero, especially for lowly-expressed genes, which could potentially affect the performance of spatial reconstruction. This issue could be addressed by employing preprocessing techniques for RNA-seq data, such as imputation or smoothing, which are accessible as functions implemented in existing R packages for transcriptome data analyses. The third is an accuracy measure for the reconstructed 3D gene expression patterns. RNA tomography based on 1D tomo-seq data along three orthogonal axes may not always accurately reconstruct any given gene expression distribution. For instance, it is impossible to reconstruct a distribution where a gene is expressed at two spots along a diagonal of the cube because infinite feasible solutions can fit the marginal distributions defined by the corresponding tomo-seq data. Currently, we are developing a method to evaluate identifiability for reconstructed patterns. The fourth is the support for the tomo-seq data prepared through sampling methods other than sampling along three orthogonal axes. A previous study provide a promising lead: Schede *et al*. successfully reconstructed 2D spatial gene expression from 1D tomo-seq data obtained from three consecutive sections secondary sliced with stripes at different angles [8]. We currently speculate that 1D tomo-seq data sampled along at least six axes enable us to accurately reconstruct any spatial distribution. The fifth is the integration of tomo-seq data with single-cell and spatial transcriptome data. For example, inference of cellular localization has been achieved by integrating single-cell RNA-seq data with landmark gene expression patterns determined by *in situ* hybridization experiments [39]. Such strategy can also employ 3D spatial expression patterns reconstructed by tomoseqr as landmarks. In addition, tomo-seq data might be integrated with 2D expression patterns derived from spatial transcriptomics techniques [40], enabling a more accurate reconstruction of 3D spatial expression patterns. The sixth is the enhancement of the functionality of *Image viewer* to allow simultaneous display of the spatial expression distributions of multiple genes. This feature would be particularly useful for morphological analyses that compare genes of interest with marker genes (e.g., tubulin beta III-positive neuronal tissue, alpha-actin-positive skeletal muscle, or cardiac myosin-positive heart).

Our evaluation showcases tomoseqr’s ability to process large datasets with computational efficiency, making it a valuable tool for exploring the complex spatial organization of gene expression in biological tissues. By offering an accurate and user-friendly approach to analyzing tomo-seq data, tomoseqr represents a significant advancement in the field of spatial transcriptomics, paving the way for new insights into developmental biology, disease mechanisms, and beyond.

## Supporting information

S1 Video Interface of *masker*. (MP4)

S2 Video Interface of *Image viewer* (2D). (MP4)

S3 Video Interface of *Image viewer* (3D). (MP4)

S4 Video Reconstruction result of *Djf1* (3D). (MP4)

S5 Video Reconstruction result of *DjNp19* (3D). (MP4)
